# Clinical and laboratory characteristics but not response to treatment can distinguish children with definite growth hormone deficiency from short stature unresponsive to stimulation tests

**DOI:** 10.3389/fendo.2024.1288497

**Published:** 2024-03-01

**Authors:** Maria Andrea Lanzetta, Eva Dalla Bona, Gianluca Tamaro, Viviana Vidonis, Giada Vittori, Elena Faleschini, Egidio Barbi, Gianluca Tornese

**Affiliations:** ^1^ Department of Medicine, Surgery and Health Sciences, University of Trieste, Trieste, Italy; ^2^ Institute for Maternal and Child Health IRCCS “Burlo Garofolo”, Trieste, Italy

**Keywords:** endocrinologic diseases, stimulation tests, epidemiology, growth hormone deficiency, short stature

## Abstract

**Introduction:**

It has been proposed that not all children with short stature displaying an inadequate response to tests for growth hormone (GH) secretion truly suffer from GH deficiency (GHD). Only children with a monogenic cause of GHD or an identifiable combined hormonal deficiency or anatomical anomaly in the hypothalamic-pituitary axis should be considered definite GHD (dGHD). The remaining patients can be defined as a separate group of patients, “short stature unresponsive to stimulation tests” (SUS). The aim of this proof-of-concept study, was to assess whether SUS patients treated with rhGH exhibit any differences compared to GHD patients undergoing the same treatment.

**Methods:**

Retrospective analysis on 153 consecutive patients with short stature and pathological response to two GH stimulation tests. Patients with dGHD were defined as those with a clear genetic or anatomical hypothalamic-pituitary anomaly, as well as those with combined pituitary hormone deficiencies and those with a known insult to the hypothalamic-pituitary axis (i.e. total brain irradiation) (n=38, 25%); those without any of the previous anomalies were defined as SUS (n=115, 75%).

**Results:**

At diagnosis, dGHD and SUS populations did not differ significantly in sex (F 32% vs 28%, p=0.68), age (11.9 vs 12.1, p=0.45), height SDS at diagnosis (-2.2 vs. -2.0, p=0.35) and prevalence of short stature (height <-2 SDS) (56% vs 51%, p=0.45). IGF-1 SDS were significantly lower in dGHD (-2.0 vs -1.3, p<0.01). After 1 year of treatment, the prevalence of short stature was significantly reduced in both groups (31% in dGHD vs. 21% in SUS, p<0.01) without any significant differences between groups (p=0.19), while the increase in IGF-1 SDS for bone age was greater in the dGHD category (+1.9 vs. +1.5, p<0.01), with no further difference in IGF-1 SDS between groups. At the last available follow-up, 59 patients had reached the near adult height (NAH) and underwent retesting for GHD. No differences in NAH were found (-0.3 vs. -0.4 SDS, 0% vs. 4% of short stature). The prevalence of pathological retesting was higher in dGHD (60% vs. 10%, p<0.01) as well as of overweight and obesity (67% vs. 26%).

**Conclusion:**

Stimulation tests and the equivalent benefit from rhGH therapy, cannot distinguish between dGHD and SUS populations. In addition, lower IGF-1 concentrations at baseline and their higher increase during treatment in dGHD patients, and the lack of pathological retesting upon reaching NAH in SUS patients, are facts that suggest that deficient GH secretion may not be the cause of short stature in the SUS studied population.

## Introduction

Childhood growth hormone deficiency (GHD) is a rare endocrine disorder characterized by inadequate secretion of growth hormone (GH) from the pituitary gland, with an estimated prevalence between 1 in 3,500 and 1 in 10,000 children (1). It usually results in short stature and can lead to significant physical and psychosocial challenges for affected children. Conventionally, the diagnosis of GHD is confirmed through stimulation tests that evaluate the ability of the pituitary gland to produce an appropriate GH response (2).

However, the accurate diagnosis of GHD in children who present with short stature or slow growth remains a diagnostic dilemma for clinicians, since several issues have been raised regarding the reliability of stimulation tests (3, 4). As a matter of fact, it has been calculated that the probability of a true positive result for a stimulation test in a child with short stature is about 1 in 36 cases (5).

It has been previously proposed that not all children with short stature displaying an inadequate response to tests for GH secretion truly suffer from GHD. Instead, amongst these, solely children with an identifiable monogenic cause of GHD or an identifiable functional or anatomical anomaly in the hypothalamic-pituitary axis should be considered definite GHD (dGHD). The remaining patients could be defined as having a “Short stature Unresponsive to Stimulation tests” (SUS) (4), rather than “idiopathic GHD” (6). In other words, SUS patients may well benefit from GH treatment even if GH deficiency is not the certain cause of their growth failure, which could be related to other causes, e.g. genetic short stature.

This study aims to present a proof of concept for the definition of SUS by providing a comprehensive analysis of the clinical and laboratory features in children diagnosed with GHD and treated with recombinant human GH (rhGH). To the best of our knowledge, no other authors have analyzed the differences between the two populations.

## Materials and methods

We conducted a retrospective study at the Institute for Maternal and Child Health IRCCS “Burlo Garofolo” in Trieste, Italy, a tertiary hospital and research institute that serves as a pediatric referral center for the province of Trieste, and as a national reference hospital.

All records of children and adolescents diagnosed with GHD from July 5^th,^ 2014 to March 31^st,^ 2022 were reviewed. Since July 5^th^, 2014, according to Italian regulation (7), GHD is defined by at least one of the following clinical-auxological parameters:

- *Criterion a)* Height ≤ -3 SDS (standard deviation score); or
- *Criterion b)* Height ≤ -2 SDS and growth velocity/year ≤ -1.0 SDS for age and sex, assessed at a distance of at least 6 months, or a decrease in height of 0.5 SDS/year in children older than two years; or
- *Criterion c)* Height ≤ -1.5 SDS compared to the genetic target and growth velocity/year ≤ -2 SDS or ≤ -1.5 SDS after 2 consecutive years; or
- *Criterion d)* Growth velocity/year ≤ -2 SDS or ≤ -1.5 SDS after two consecutive years, even in the absence of short stature and after excluding other pathological conditions as the cause of growth deficiency; or
- *Criterion e)* Hypothalamic-pituitary malformations/lesions demonstrated by neuro-radiological imaging;

and a GH response <8 ng/mL in two pharmacological tests performed on different days. One of the two tests can be growth hormone-releasing hormone (GHRH)+arginine, and in this case, GHD is defined as a GH response <20 ng/mL.

Definite GHD (dGHD) was defined (4) when at least one of the following criteria was present: genetic diagnosis of isolated GHD (pathogenic mutation in *GH1*, *GHRHR* or *RNPC3* genes); combined pituitary hormone deficiencies (CPHD); presence of abnormalities within the hypothalamus-pituitary axis at magnetic resonance imaging (MRI); acquired damage (such as brain trauma, central nervous system infection, tumors of the hypothalamus or pituitary, radiation therapy, infiltrative diseases). All the other patients were considered as SUS.

The height and BMI SDS were determined by employing Growth Calculator 3 Software using WHO reference charts (8, 9), which were chosen over national reference charts to avoid the underestimation of overweight and obesity (10). Short stature was defined as height <-2 SDS. Overweight was defined as BMI SDS between 1 and 2 SDS and obesity as BMI SDS >2. The IGF-1 SDS were determined according to bone age (11). Differences (Δ) in variables were calculated compared to baseline values.

When near adult height (NAH) [defined as a height velocity of <2 cm/year, an individual growth curve showing asymptotic growth toward adult height, and bone age of ≥15 years of age (12)] was reached, retesting of GH secretion was performed with growth hormone-releasing hormone (GHRH)+arginine test, after at least 1 month of rhGH treatment suspension. Persistent GHD was defined as a GH response <19 ng/mL. Stimulation tests were performed according to protocols, as previously described (13).

Brain MRI was performed in all individuals before rhGH treatment started. Anomalies of the pituitary gland at MRI were defined as such by expert pediatric radiologists and through comparison with relevant pediatric literature (14, 15). Pituitary stalk agenesis, ectopic posterior pituitary, and pituitary stalk interruption syndrome are indeed highly specific findings for GHD (16). Empty sella and pituitary hypoplasia instead, while also found in the general population, were considered relevant when in association with hormonal deficit (17).

Since normal MRI imaging reasonably excludes GH1, GHRHR, or RNPC3 mutations (18–20), genetic testing for these genes was not performed routinely in our cohort. According to our clinical practice, indications for genetic testing were: familial short stature, disproportionate short stature, facial dysmorphisms, and skeletal abnormalities (e.g. Madelung deformity, brachydactyly). When performed, genetic analysis was limited to next-generation sequencing of genes known to be associated with short stature: SHOX, NPR2, CNP, IHH, ACAN, PAPPA2, FGFR3, STAT5B, GHR, GH1, IGF1, IGF1R, IGFALS, GHSR.

The “G2 clinico” platform (management system specialist activities) was employed to access all patients’ data. Information retrieved included gender, target height, criteria to perform GH stimulation tests, type of tests performed and GH peaks, presence of genetic mutations, presence of other pituitary deficiencies, anomalies at MRI, presence of acquired pituitary damage; at diagnosis, after 1 year of treatment and at last follow-up visits: age, IGF-1, bone age (according to Greulich & Pyle) (21), rhGH dose.

Ethical Committee approval was not requested since General Authorization to Process Personal Data for Scientific Research Purposes (Authorization no. 9/2014) declared that retrospective archive studies that use ID codes, preventing the data from being traced back directly to the data subject, do not need ethics approval (22). According to the Research Institute policy informed consent was signed by parents at the first visit, in which they agreed that “clinical data may be used for clinical research purposes, epidemiology, study of pathologies and training, with the objective of improving knowledge, care and prevention”.

All statistical analyses were conducted with JMP™ (version 16.1.0, SAS Institute Inc., Cary, NC, United States). Data are presented as median and interquartile ranges (IQRs). Mann-Whitney rank-sum tests and Two-tailed Fisher exact tests were performed to evaluate the relations between variables. Wilcoxon signed-rank test was performed to check whether the differences between paired data were statistically significant. Single-linear regression and multivariate logistics regressions were carried out to study associations between a dichotomous outcome and one or more independent variables. A p-value <0.05 was considered statistically significant.

## Results

**Figure 1:**
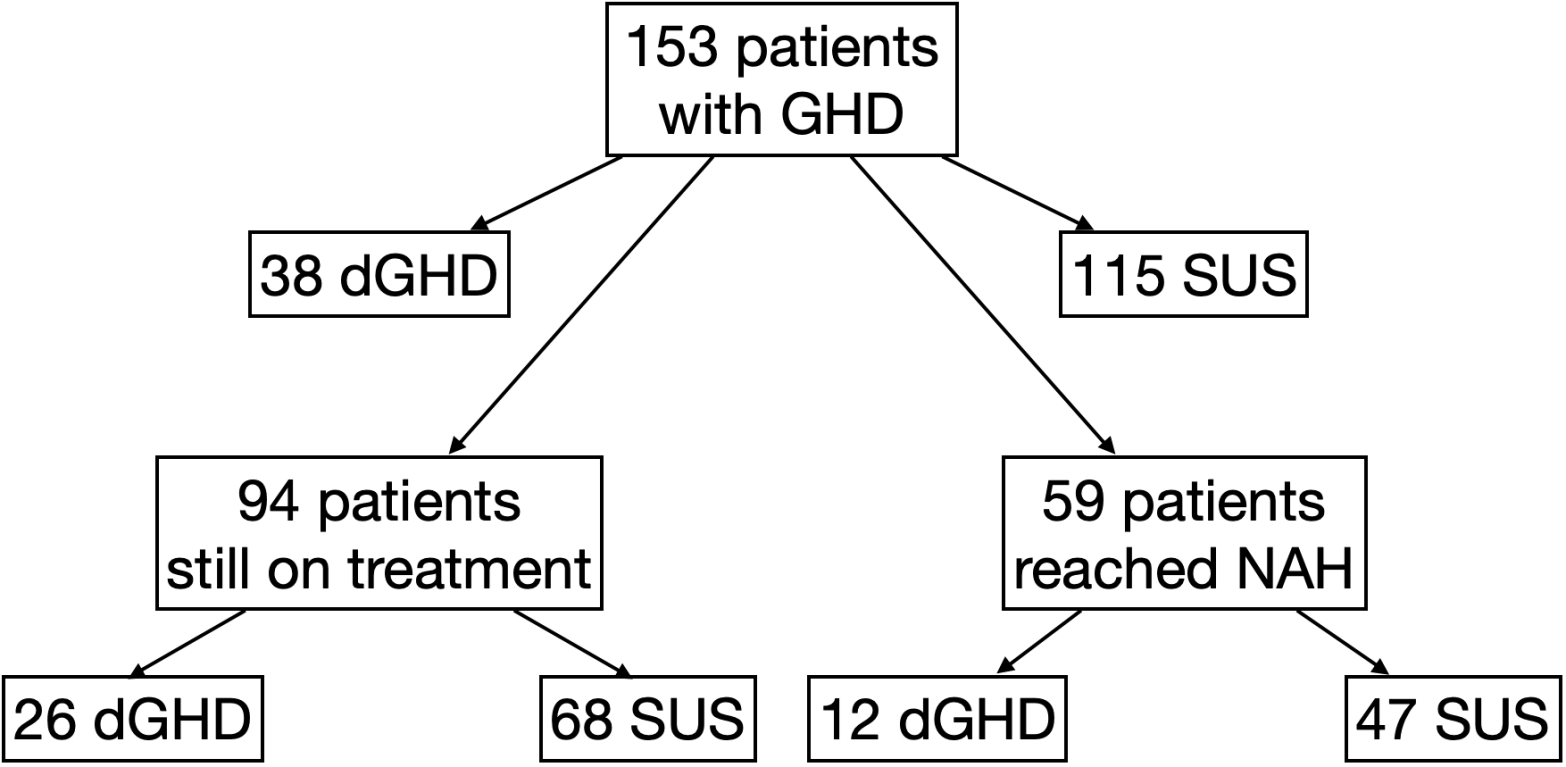
Flow-chart explaining the division to different groups of the study. dGHD, definite growth hormone deficiency; GHD, growth hormone deficiency; SUS, short stature unresponsive to stimulation tests.

We collected data on 153 consecutive patients (44 females) with a median age at diagnosis of GHD of 12.0 years (IQR 9.6;13.5), 59% pre-pubertal. The first stimulation tests were performed with arginine (n=149), GHRH+arginine (n=3), or glucagon (n=1); the second stimulation tests were performed with insulin (n=115), arginine (n=26), clonidine (n=11) or GHRH+arginine (n=1). Overall, 38 individuals were identified as having dGHD (n=32 with MRI abnormalities [n=18 reduced pituitary volume; n=7 empty sella; n=4 pituitary stalk interruption syndrome (PSIS); n=2 pituitary stalk thickening; n=1 pituitary agenesis]; n=5 with CPHD, 3 of whom had MRI abnormalities; n=2 with genetic diagnosis [pathogenetic mutations in *GH1* gene]; n=2 with acquired damage secondary to brain radiotherapy). The remaining 115 individuals were identified as SUS (**Figure 1**). 16 children (10%) with a known genetic abnormality (unrelated to GHD) were included in the SUS category (4 with muscular dystrophies; 3 with metabolic disorders such as Kearns-Seyre syndrome, GLUT1 deficiency, and hyperinsulinism/hyperammonemia syndrome; 4 with isolated genetic abnormalities such as Xp22.3 duplication, 13q21.2 deletion, 20q13.33 deletion and 2q37 deletion; 1 with tricho-entero-hepatic syndrome and 4 with syndromes known to affect growth such as ACAN mutation, Mazzanti syndrome and MIRAGE syndrome). All other patients were otherwise healthy. Clinical-auxological parameters for GHD diagnosis and dGHD criteria are presented in **Table 1**.

**Table 1:** GHD diagnostic criteria according to the Italian regulation: comparison of the whole cohort, GHD and SUS population and prevalence of the proposed dGHD diagnostic criteria in the dGHD population.

	Total	dGHD	SUS	p
GHD diagnosis - clinical-auxological parameters				
Height ≤ -3 SDS	8%	13%	7%	0.23
Height ≤ -2 SDS and growth velocity/year ≤ -1.0 SDS, or a decrease in height of 0.5 SDS/year	53%	45%	56%	0.24
Height ≤ -1.5 SDS compared to the genetic target and growth velocity/year ≤ -2 SDS or ≤ -1.5 SDS after 2 consecutive years	29%	18%	32%	0.10
Growth velocity/year ≤ -2 SDS or ≤ -1.5 SDS after two consecutive years	77%	71%	79%	0.30
Hypothalamic-pituitary malformations/lesions at imaging	20%	82%	0%*	**<0.01**
dGHD criteria				
Genetic diagnosis of isolated GHD	1%	5%	0%	
Multiple pituitary combined deficiencies	3%	13%	0%	
Hypothalamus-pituitary axis abnormalities at MRI	21%	84%	0%	
Acquired damage	1%	5%	0%	

SDS, standard deviation score. Significant p values in bold.

* “Hypothalamic-pituitary malformations/lesions at imaging” were one of the criteria that defined dGHD.

**Figure 2:**
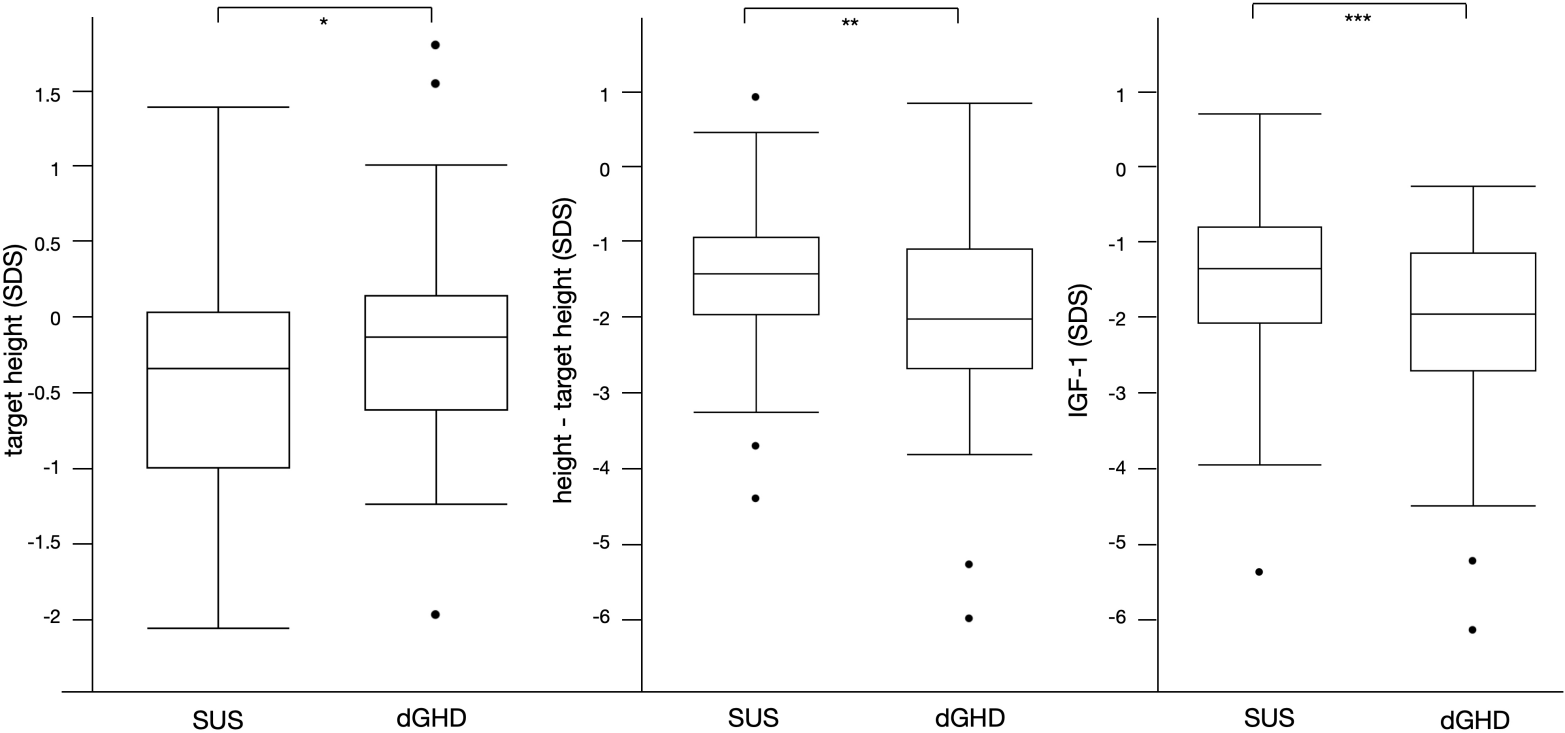
Significant differences between SUS and dGHD at baseline: target height SDS (* p=0.04), height-target height SDS (**p=0.01), IGF-1 SDS (***p<0.01).

The clinical and laboratory characteristics of the entire cohort and the 2 groups at baseline are reported in **Table 2**. While no difference was found in height at diagnosis between dGHD and SUS (-2.2 vs. -2.0 SDS, p=0.35), target height in SDS was lower in SUS than in dGHD (-0.3 vs. -0.1, p=0.04) and the difference between height and target height in SDS was higher in dGHD than in SUS (-2.1 vs. -1.4, p=0.01) (**Figure 2**).

**Table 2:** Clinical and laboratory characteristics at baseline.

	Total	dGHD	SUS	p
N (%)	153 (100%)	38 (25%)	115 (75%)	
Sex (female, %)	29%	32%	28%	0.68
Target height (SDS)	-0.3 (-0.8;0.2)	-0.1 (-0.6;0.2)	-0.3 (-1.0;0.1)	**0.04**
Age (years)	12.0 (9.6;13.5)	11.9 (8.0;13.4)	12.1 (9.9;13.6)	0.45
Pre-pubertal (%)	59%	74%	55%	**0.03**
Height (SDS)	-2.0 (-2.5;-1.4)	-2.2 (-2.8;-1.2)	-2.0 (-2.5;-1.4)	0.35
Height – TH (SDS)	-1.5 (-2.2;-1.0)	-2.1 (-2.7;-1.2)	-1.4 (-2.0;-0.9)	**0.01**
Short stature (%)	52%	56%	51%	0.45
BMI (SDS)	-0.1 (-1.0;1.1)	0.2 (-0.8;1.4)	-0.1 (-1.1;1.0)	0.43
Overweight/obese (%)	18%/9%	26%/13%	17%/8%	0.19
Bone age (years)	11.0 (8.0;12.5)	11.0 (6.0;12.6)	11.0 (8.5:12.5)	0.92
Bone age-chronological age (years)	-1.4 (-2.3;-0.5)	-1.1 (-1.8;-0.3)	-1.5 (-2.4;-0.7)	0.08
GH peak 1^st^ test (ng/ml)	4.5 (3.1;6.0)	3.8 (2.1;5.2)	4.8 (3.5;6.1)	**<0.01**
GH peak 1^st^ test – arginine only (ng/ml)	4.6 (3.0;6.0)	3.8 (2.1;5.2)	4.8 (3.6;6.0)	**<0.01**
GH peak 1^st^ test <3 ng/mL –arginine only (%)	23%	37%	19%	**0.02**
GH peak 2^nd^ test (ng/ml)	2.8 (1.6;4.5)	3.2 (1.8;4.5)	2.5 (1.5;4.4)	0.35
GH peak 2^nd^ test – insulin only (ng/ml)	2.3 (1.3;4.0)	3.5 (1.3;5.2)	2.2 (1.3;3.9)	0.06
GH peak 2^nd^ test <3 ng/mL – insulin only (%)	63%	41%	70%	**<0.01**
IGF-1 (SDS)	-1.4 (-2.2;-0.9)	-2.0 (-2.8;-1.4)	-1.3 (-2.1;-0.8)	**<0.01**
IGF-1 <0 SDS (%)	94%	100%	92%	0.07
IGF-1 <-1.5 SDS (%)	52%	58%	35%	**0.02**
IGF-1 <-2 SDS (%)	31%	48%	25%	**0.01**
Genetic analysis performed	29%	50%	21%	**<0.01**
rhGH dose (mcg/kg/day)	25.1 (24.0;26.6)	25.4 (24.0;27.0)	25.0 (23.8;26.5)	0.17

BMI, body mass index; GH, growth hormone; IGF-1, insulin-like growth factor 1; rhGH, recombinant human growth hormone; SDS, standard deviation score; TH, target height. Significant p values in bold.

There was a higher prevalence of pre-pubertal children in dGHD than in SUS (74% vs. 55%, p=0.03), although no significant differences were found in age at diagnosis (11.9 vs. 12.1 years, p=0.45). Individuals with dGHD had lower GH peaks at the first stimulation tests with arginine (3.8 vs. 4.8 ng/mL, p<0.01), with a higher prevalence of GH peak <3 ng/mL (37 vs. 19%, p=0.02), although SUS had a higher prevalence of GH peak <3 ng/mL at second stimulation tests with insulin (70% vs. 41%, p<0.01).

At diagnosis, dGHD had lower IGF-1 in SDS (-2.0 vs -1.3, p<0.01) (**Figure 2**), with a higher prevalence of IGF-1 <-1.5 SDS (58% vs. 35%, p=0.02) and <-2 SDS (48% vs. 25%) (**Table 2**). However, the IGF-1 <-1.5 SDS cut-off had a sensitivity was 66% and specificity 58% for dGHD, and the IGF-1 <-2 SDS cut-off had a sensitivity was 0%, and specificity 100%.

All patients reached at least 1 year of follow-up, and both GHD and SUS benefitted from rhGH therapy. Indeed, median increase in height SDS after 1 year of treatment was 0.5 in both groups. The difference between height and target height in dGHD compared to SUS persisted (-1.6 vs. -0.9 SDS, p=0.02). Although the height gain was similar in the two groups, dGHD exhibited a greater increase in IGF-1 compared to baseline was found in SUS (+1.9 vs. +1.5 SDS, p<0.01) (**Supplementary Table 1**).

Among the entire cohort, 94 individuals (61%) were still on treatment (26 dGHD; 68 SUS) (**Figure 1**); the median age at the last available follow-up was 13.5 years (IQR 11.1;15.1) with a median length of treatment of 3.2 years (IQR 1.4;4.1). At the last follow-up visit, a greater difference between height and target height was still found in dGHD compared to SUS (-1.2 vs. -0.4 SDS, p=0.02), and a higher rhGH dose was used in dGHD (29.8 vs. 27.8 mcg/kg/day, p=0.03) (**Supplementary Table 2**).

**Figure 3:**
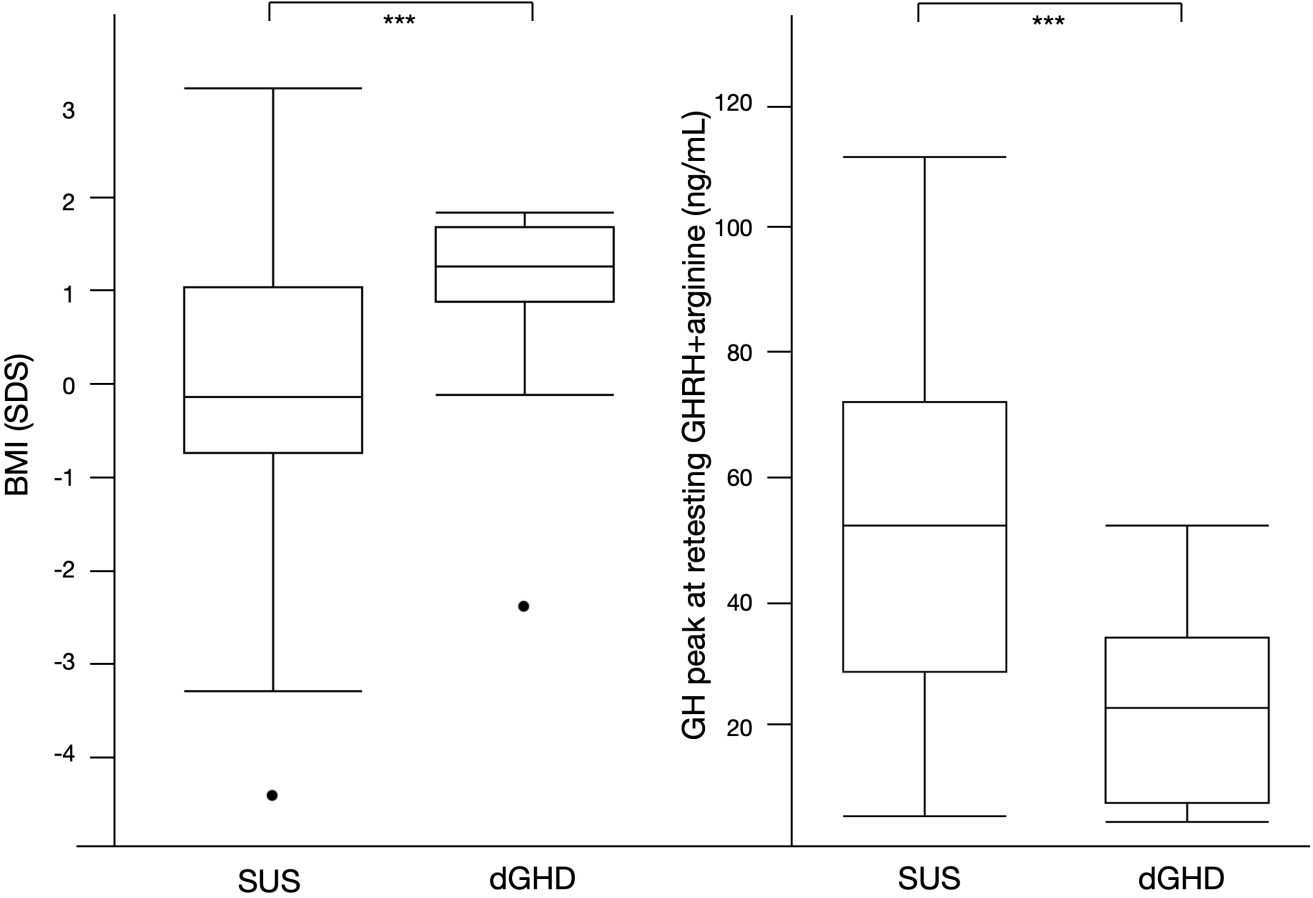
Significant differences between SUS and dGHD at the last follow-up visit for individuals who reached near adult height (n=59): BMI SDS and GH peak at retesting with GHRH+arginine (*** p<0.01).

Overall, 59 individuals reached NAH and ended rhGH treatment (39%; 12 dGHD; 47 SUS) (**Figure 1**), at a median age of 17.2 years (IQR 16.0;18.1) with a median length of treatment of 4.1 years (IQR 3.1;4.7). Those with dGHD had a higher prevalence of pathological retesting (40% vs. 10%, p<0.01) with a lower GH peak at GHRH+Arginine test (23.4 vs. 52.7 ng/mL, p<0.01) (**Figure 3**, **Table 3**). Moreover, dGHD individuals with pathological retesting (2 with PSIS and 2 with CPHD) had significantly lower IGF-1 SDS (median -2.4 SDS [IQR -4.1;-1.5]) compared to those with normal retesting (median -1.0 [-1.9;-0.6]) (3 with empty sella, 2 with pituitary hypoplasia and 1 with a pathogenic mutation in the GH1 gene). A greater BMI SDS was found in dGHD compared to SUS (1.3 vs. -0.1, p<0.01) with a higher prevalence of overweight and obesity (67% vs. 26%, p<0.01) (**Figure 3**, **Table 3**). No increased prevalence of CPHD, hypothalamus-pituitary axis abnormalities at MRI, or acquired damage were found in dGHD individuals with overweight and obesity at the last follow-up; moreover, no limited subjects’ mobility was reported. Although these children had a higher BMI already at baseline (1.22 vs 0.14 SDS) this difference did not reach statistical significance (p=0.06). All dGHD with pathological retesting were overweight/obese (100%) compared to 50% of those with normal retesting and 26% of SUS both with or without pathological retesting (p<0.01). Peak GH in pathological retesting was correlated with IGF-1 SDS (r^2^ 0.50, p=0.04), but not with BMI SDS (r^2^ 0.01, p=0.81). The median IGF-1 of dGHD patients with pathological retesting was -2.4 SDS (-4.1;-1.5).

**Table 3:** Clinical and laboratory characteristics at the last follow-up visit for individuals who reached near adult height (n=59).

	Total	dGHD	SUS	p
N (%)	59 (100%)	12 (20%)	47 (80%)	
Sex (female, %)	20%	20%	20%	0.96
Age (years)	17.2 (16.0;18.1)	17.4 (16.1;18.3)	17.1 (16.0;18.1)	0.64
Length of treatment (years)	4.1 (3.1;4.7)	4.2 (3.4;4.5)	3.8 (3.1;4.7)	0.52
NAH (SDS)	-0.5 (-1.0;0.3)	-0.3 (-1.0;0.3)	-0.4 (-1.0;0.3)	0.70
NAH – TH (SDS)	0.0 (-0.6;0.4)	-0.5 (-1.2;0.3)	0.0 (-0.3;0.4)	0.11
Short stature (%)	3%	0%	4%	0.46
Δ Height (SDS)	1.4 (0.9;1.9)	1.5 (0.8;1.8)	1.3 (0.9;1.9)	0.93
BMI (SDS)	0.3 (-0.6;1.3)	1.3 (-0.9;1.7)	-0.1 (-0.7;1.0)	**<0.01**
Overweight/obese (%)	27%/7%	67%/0%	17%/9%	**<0.01**
Δ BMI (SDS)	-0.1 (-0.6;0.5)	0.1 (-0.4;0.7)	-0.2 (-0.7;0.5)	0.35
Bone age (years)	16.0 (15.0;16.5)	16.3 (15.3-17.0)	15.5 (15.0-16.5)	0.15
Bone age-chronological age (years)	-1.3 (-2.2;-0.1)	-1.1 (-2.0;0.0)	-1.3 (-2.4;-0.1)	0.57
Δ bone age/Δ chronological age	0.9 (0.7;1.3)	0.9 (0.6;1.2)	0.9 (0.7;1.3)	0.48
IGF-1 (SDS)	-1.2 (-1.8;-0.7)	-1.2 (-2.3;-0.3)	-1.2 (-1.6;-0.7)	0.75
Δ IGF-1 (SDS)	0.2 (-0.4;1.3)	0.6 (0.1;1.4)	0.2 (-0.5;1.3)	0.27
rhGH dose (mcg/kg/day)	28.4 (26.0;32.9)	29.1 (27.3;32.6)	28.3 (25.0;32.9)	0.39
Pathological retesting (%)	16%	40%	10%	**<0.01**
GH peak at retesting test (ng/ml)	49.1 (25.4;71.7)	23.4 (7.8;34.6)	52.7 (29.9;72.6)	**<0.01**

BMI, body mass index; GH, growth hormone; IGF-1, insulin-like growth factor 1; NAH, near adult height; rhGH, recombinant human growth hormone; SDS, standard deviation score; TH, target height; Δ BMI, difference between BMI at last follow-up compared to baseline; Δ bone age/Δ chronological age, difference between bone age at last follow-up compared to baseline over difference between chronological age at last follow-up compared to baseline; Δ height, difference between height at last follow-up compared to baseline; Δ IGF-1, difference between IGF-1 at last follow-up compared to baseline. Significant p values in bold.

**Figure 4:**
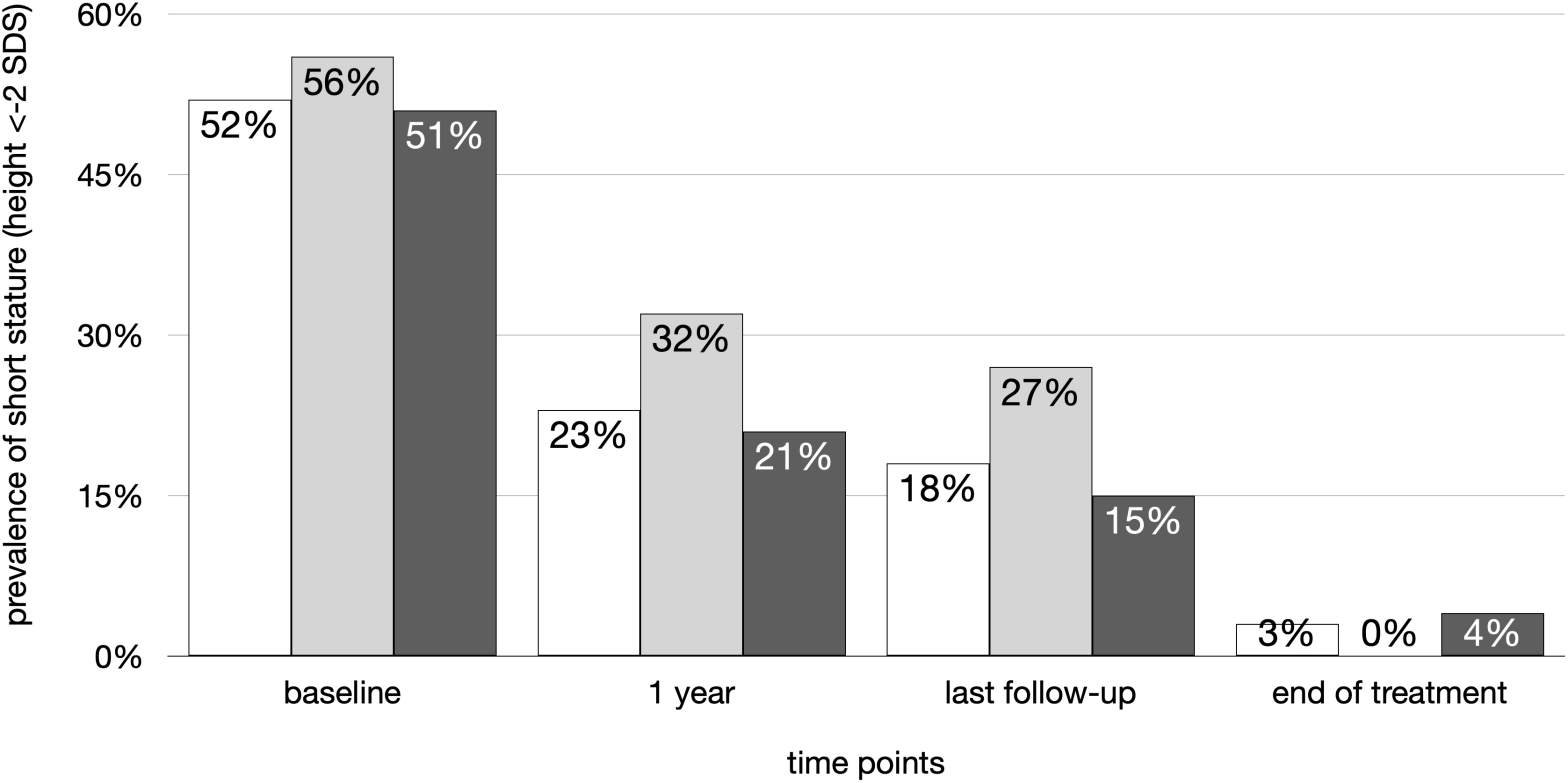
Prevalence of short stature (height < -2 SDS) in the overall cohort (white), dGHD (light grey) and SUS (dark grey) at different follow-up timepoints: at baseline, after 1 year of follow-up, at last visit and at the end of rhGH therapy.

While at baseline 52% had short stature (<- 2 SDS), after 1 year of treatment the rate decreased to 23% and 18% at the last follow-up visit or 3% at the end of rhGH treatment (p<0.01), with no significant differences between dGHD and SUS (**Figure 4**). The increase in height SDS was significant from baseline to 1 year of treatment and to the end of treatment both in dGHD (baseline -1.7 SDS [-2.3;-1.0]; 1 year -1.2 SDS [-1.5;-0.4]; end -0.3 SDS [-1.0;0.3]; p<0.01) and in SUS (baseline -1.9 SDS [-2.3;-1.3]; 1 year -1.2 SDS [-1.9;-0.7]; end -0.5 SDS [-1.0;0.3]; p<0.01).

Overall, 44 individuals performed NGS for genes causative of short stature, 25/115 among SUS (21%), and 19/38 among dGHD (50%) (p<0.01).

## Discussion

In this retrospective study, we comprehensively analyzed 153 patients diagnosed with GHD according to auxological and laboratory parameters and treated with rhGH to identify possible differences supporting the definition of SUS as a proof of concept. As a matter of fact, this study highlighted some significant differences between patients who had a definite and identifiable monogenic, functional, or anatomical cause of GH deficiency (dGHD) and those who did not (SUS).

The main findings of this retrospective study were that individuals identified as dGHD had lower IGF-1 concentrations at baseline, with a higher increase after 1 year of treatment, and had a higher prevalence of pathological retesting and overweight/obesity at the end of treatment. Individuals labeled as SUS had a lower target height and a greater difference between height and target height at diagnosis, after 1 year of treatment, and at the last available follow-up visit for those still on treatment. Nevertheless, results in terms of first-year and final responses were similar between SUS and dGHD.

As could be expected (4), GH stimulation tests were found to be of no aid in discriminating the two categories. Indeed, while GH peak was lower in dGHD at the first stimulation test, with a higher prevalence of “severe” GHD, the opposite was found at the second stimulation test. GH stimulation tests, though useful for the clinician to find some bearings in the *mare magnum* of the causes of short stature, have in fact long been known to be an imperfect means of diagnosis of GHD, displaying problems with reproducibility and yielding potential false positive or false negative results (3, 23, 24). For instance, pathological stimulation tests have been found in conditions where a problem in GH secretion is not supposed to be the cause of short stature (such as Turner syndrome, Noonan syndrome, or *SHOX* deficiency) (25–27). Similarly, we did not find any significant difference in height SDS at diagnosis between the two groups that could help in differentiating a true GHD from other causes of short stature (28, 29).

On the contrary, some major differences were in line with the etiopathogenesis of GHD: lower IGF-1 at baseline and greater increase during treatment, higher prevalence of persistent GHD at retesting, and higher BMI when they reached NAH.

With regards to IGF-1, their concentrations were lower at baseline in dGHD compared to SUS; however, due to a significant increase in dGHD patients after the first year of treatment, differences in IGF-1 SDS did not persist at 1 year or the end of treatment. Since IGF-1 is secreted by the liver when stimulated by GH, lower concentrations of IGF-1 at baseline and normalization after treatment are consistent with a differentiation between dGHD and SUS. Many IGF-1 SDS cut-offs have been proposed for the diagnosis of GHD (30, 31) and dGHD had a higher prevalence of IGF-1 <-1.5 or <-2 SDS in our cohort; however, we do not propose these cut-offs as diagnostic criteria in discriminating dGHD from SUS, but more as supportive factors. Indeed, it is already known that they suffer from low sensitivity and specificity in GHD diagnosis (13, 32), therefore their values should always be interpreted in combination with other clinical and biochemical parameters.

The majority of our patients (59%) were pre-pubertal at diagnosis, with a higher prevalence of pre-pubertal children in dGHD than in SUS. This may be partially explained by the presence, in the dGHD cohort, of patients with CPHD, which is known to be associated with a delay in pubertal development (33). Amongst all pre-pubertal patients, solely 6 (4%) had delayed puberty, defined as the absence of thelarche by 13 years of age in females or the absence of an increase in testicular volume >4 mL by 14 years of age in males. Interestingly, all patients with delayed puberty were males. It has been suggested that sex steroid priming may be useful in pre-pubertal patients of peri-pubertal age, reducing the overdiagnosis of children with constitutional delay of growth and puberty (CDGP) as GHD patients. However, presently it is not recommended by our national guidelines and there are no standardized protocols for sex steroid administration in this type of patient, particularly concerning sex steroid dose and timing of supplementation (34, 35). Furthermore, some authors have suggested that sex steroid priming may lead to an underdiagnosis of GHD patients, due to a temporary and unsustained increase in GH secretion (36). For these reasons, sex steroid priming was not performed.

Childhood-onset GHD is usually retested after the achievement of NAH to verify whether they need to continue rhGH treatment, and a rate ranging from 20 to 87% percent of individuals have been found to have normal GH secretion (37). While GH stimulation tests remain an imperfect means of defining pediatric GH deficiency, a deficient response to insulin or arginine+GHRH stimulus is highly specific for GHD in adults (38–40). The wide difference in positive retesting in pediatric GHD in the literature suggests that the GHD definition in the pediatric age is a sort of umbrella term, including different conditions. Remarkably, in our cohort, we found that almost all of SUS had normal retesting. On the contrary, more than half of dGHD had the confirmation of a pathological GH secretion and continued rhGH treatment. These findings are consistent with the reported rates of persistent GHD in patients with known pituitary abnormalities (such as ectopic posterior pituitary or pituitary hypoplasia) ranging from 27 to 66% (41, 42). For example, in some genetically determined GHD (such as GHRHR mutations) GH secretion may be reduced and not completely absent (43). Indeed, one of our dGHD individuals with normal retesting had a pathogenic mutation in the GH1 gene. We therefore believe that normal retesting does not exclude the dGHD diagnosis.

It has been reported that GHD is associated with mild to moderate truncal obesity, mostly in adults (44, 45), and mild to moderate overweight is usually thought to be a feature of GHD also in children (46); however, children with GHD have been shown to have average BMI, with no differences between organic and idiopathic GHD (46, 47), therefore only the pattern of fat distribution should be considered in the clinical diagnosis of GHD. In our cohort, we did not evaluate fat mass; however, no significant differences were noticed in BMI between dGHD and SUS at baseline or after 1 year of treatment. However, at the end of treatment, dGHD had a greater BMI compared to SUS and a higher prevalence of overweight/obese adolescents. Interestingly, all dGHD with pathological retesting were overweight/obese, compared to half of those with normal retesting or a quarter of those with SUS. While it has been suggested that the GHRH+arginine stimulation test is highly dependent on BMI (48), in our cohort peak GH was correlated with IGF-1 SDS in pathological retesting, with no correlation with BMI SDS.

A significant increase in BMI in non-overweight children after 2 years of rhGH treatment (47) and in adults after 3 years of therapy (49) has been reported, however, no studies have ever evaluated BMI at the end of childhood GHD so far. It should be noted that 40% of dGHD with pathological retesting presented multiple pituitary hormone deficits requiring hydrocortisone supplementation. A single patient instead presented multiple pituitary hormone deficiency with central hypothyroidism. In all cases, however, hormone supplementation was optimized and unlikely to contribute to weight gain. A possible explanation could be that the increase in BMI is a characteristic of GHD that is evident only after childhood and only in severe GHD (i.e. persistent GHD after childhood), although further studies are necessary to explore this issue.

An interesting finding was that target height SDS was significantly lower in children with SUS than in those with dGHD. Moreover, the difference between height and target height in SDS was higher in dGHD than in SUS at baseline as well as after 1 year of treatment and at the last follow-up visit, but not at the end of treatment. These data suggest that the mechanism underlying short stature in SUS patients might not be GH deficiency, nor CDGP (50), but autosomal dominant short stature (51, 52), with mutations in genes known to influence the growth plate independently of GH, such as paracrine signaling and the composition of extracellular matrix and chondrocytes (53–55). A recent study found that among children with GHD and a family history of short stature a causative genetic mutation was found in 29%, none of them were in genes related to isolated GHD (*GH1*, *GHRHR*, or *RNPC3*) and only 13% had a genetic variant affecting GH secretion or function (*GSHR* and *OTX2)*, while mostly had a primary growth plate disorder; therefore, genetic results frequently did not correspond with the clinical diagnosis of GHD, even with faltering response to stimulation tests (56). Moreover, the same study showed that genetic causes for short stature may have an excellent response to rhGH treatment, contrary to what was thought so far about short genetic stature (56). While these results may be influenced by our still incomplete knowledge of genetic defects affecting the GH-IGF1 axis, they emphasize the hypothesis that the low response of GH during stimulation tests may be an epiphenomenon rather than the cause for short stature. Therefore, whenever feasible, pediatric endocrinologists should consider performing genetic studies as part of the routine diagnostic work-up for short stature (57).

Besides these differences, dGHD and SUS groups did not differ at baseline for sex, age, height SDS, bone age, the difference between bone and chronological age, and prescribed rhGH dose. Moreover, no significant advance in bone age during rhGH treatment has been found in dGHD or SUS (58). In particular, our data confirm the effectiveness of rhGH treatment in both groups. The first-year response to rhGH treatment, a critical determinant of the total treatment height outcome in growth disorders (59), was good in both groups, and both groups reached the target height and a normal height. Therefore, a good response to rhGH treatment is non-specific and should not be used to define the etiology of short stature (5) or to decide to whom treatment should be offered. Indeed, rhGH therapy has been found to improve both short-term and long-term height gain even in children with idiopathic short stature (ISS) (60); however, usually, supraphysiological doses of rhGH are required in ISS (61), with lower gain in height SDS compared to GHD (62), which is not the case for SUS patients in our study.

This study has limits. It is based on retrospective data collection from a single center, therefore results may be related to the local population. Moreover, although a normal MRI realistically excludes *GH1*, *GHRHR*, or *RNPC3* mutations, genetic analyses were not performed in 90 out of 115 SUS patients and not all known genes associated with GHD (for example *GHRHR* and *RNPC3*) were included in the NGS analysis, therefore some patients may have been inappropriately considered as SUS. In addition, amongst the SUS patients, 10% had a genetically determined syndrome which may have impacted GH secretion. Lastly, we had data at the end of treatment only for 59 individuals (39% of the cohort).

On the other hand, to our knowledge, this is the first study comparing dGHD and SUS children from their diagnosis to the end of rhGH treatment, showing that they are two distinct groups with differences in IGF-1 concentrations, target height, distance from target height at baseline and BMI SDS and positive retesting at the end of treatment.

In our view, SUS is not a definitive diagnosis, and a strategy to increase the rates of correct and precise diagnosis should be developed. We have suggested the distinction between the SUS and dGHD populations to help in defining the different etiologies of short stature, in a way that may keep the field open to other relevant diagnoses, such as genetic short stature, and applications of research. Moreover, it may avoid labeling children with a diagnosis that is not entirely confirmed and that entails multiple clinical sequelae over the years (e.g., the evolution of subsequent multiple pituitary hormone deficiencies, altered body composition, decreased bone mineral density, persistent GHD at retesting. The findings of this study are proof of concept of the definition of SUS: not all children with abnormal responses to GH stimulation tests have GHD. However, this does not mean that rhGH treatment is not advisable in SUS patients. On the contrary, rhGH treatment should continue to be offered to children with SUS, since results in terms of first-year and final response are similar to those of children with dGHD.

## Data availability statement

The raw data supporting the conclusions of this article will be made available by the authors, without undue reservation.

## Ethics statement

Ethical Committee approval was not requested since General Authorization to Process Personal Data for Scientific Research Purposes (Authorization no. 9/2014) declared that retrospective archive studies that use ID codes, preventing the data from being traced back directly to the data subject, do not need ethics approval. Informed consent was signed by parents at the first visit, in which they agreed that “clinical data may be used for clinical research purposes, epidemiology, study of pathologies and training, with the objective of improving knowledge, care and prevention”.

## Author contributions

ML: Data curation, Investigation, Writing – original draft. ED: Data curation, Investigation, Writing – original draft. GTa: Conceptualization, Supervision, Validation, Writing – review & editing. VV: Investigation, Writing – review & editing. GV: Investigation, Writing – review & editing. EF: Validation, Writing – review & editing. EB: Resources, Validation, Writing – review & editing. GTo: Conceptualization, Data curation, Formal analysis, Funding acquisition, Methodology, Supervision, Writing – review & editing.
