# Supplementary material for: Clinical and laboratory characteristics but not response to treatment can distinguish children with definite growth hormone deficiency from short stature unresponsive to stimulation tests
Source: Front Endocrinol (Lausanne). 2024 Mar 1;15:1288497. doi: 10.3389/fendo.2024.1288497 (PMC10940512; doi:10.3389/fendo.2024.1288497)
Supplement: Supplementary file 1 [file Table_1.pdf]

Supplementary Table 1. Clinical and laboratory characteristics after 1 year of treatment (n=153). BMI, body mass index; GH, growth hormone; IGF-1, insulin-like growth factor 1; NAH, near adult height; rhGH, recombinant human growth hormone; SDS, standard deviation score; TH, target height; Δ BMI, difference between BMI after 1 year compared to baseline; Δ bone age/Δ chronological age, difference between bone age after 1 year compared to baseline over difference between chronological age after 1 year compared to baseline; Δ height, difference between height after 1 year compared to baseline; Δ IGF-1, difference between IGF-1 after 1 year compared to baseline

	**Total**	**dGHD**	**SUS**	**p**
N (%)	153 (100%)	38 (25%)	115 (75%)	
Height (SDS)	-1.5 (-2.0;-0.9)	-1.5 (-2.1;-0.8)	-1.4 (-1.9;-0.9)	0.54
Height – TH (SDS)	-0.9 (-1.7;-0.4)	-1.6 (-2.2;-0.4)	-0.9 (-1.4;-0.4)	**0.02**
Short stature (%)	23%	32%	21%	0.19
Δ Height (SDS)	0.5 (0.2;0.9)	0.5 (0.2;0.9)	0.5 (0.2;0.9)	0.91
BMI (SDS)	-0.3 (-1.1;0.9)	0.4 (-1.3;1.5)	-0.3 (-1.0;0.7)	0.38
Overweight/obese (%)	17%/9%	23%/5%	15%/7%	0.43
Δ BMI (SDS)	-0.2 (-0.5;0.1)	-0.3 (-0.5;0.0)	-0.2 (-0.5;0.1)	0.42
Bone age (years)	12.0 (9.3;13.5)	12.0 (6.8;14.0)	12.0 (9.6;13.5)	0.94
Bone age-chronological age (years)	-1.5 (-2.3;-0.7)	-1.3 (-2.0;-0.5)	-1.5 (-2.3;-0.7)	0.31
Δ bone age/Δ chronological age	0.8 (0.4;1.4)	0.6 (0.3;1.3)	0.8 (0.4;1.4)	0.23
IGF-1 (SDS)	0.1 (-0.6;0.8)	0.0 (-0.5;0.8)	0.1 (-0.6;0.8)	0.80
Δ IGF-1 (SDS)	1.5 (0.9;2.1)	1.9 (1.3;2.8)	1.5 (0.9;1.8)	**<0.01**
rhGH dose (mcg/kg/day)	27.6 (24.8;30.7)	27.6 (24.3;32.8)	27.6 (24.9;30.0)	0.69
