# Supplementary material for: Clinical and laboratory characteristics but not response to treatment can distinguish children with definite growth hormone deficiency from short stature unresponsive to stimulation tests
Source: Front Endocrinol (Lausanne). 2024 Mar 1;15:1288497. doi: 10.3389/fendo.2024.1288497 (PMC10940512; doi:10.3389/fendo.2024.1288497)
Supplement: Supplementary file 2 [file Table_2.pdf]

Supplementary Table 2. Clinical and laboratory characteristics at the last follow-up visit for individuals who are currently on rhGH treatment (n=94). BMI, body mass index; GH, growth hormone; IGF-1, insulin-like growth factor 1; NAH, near adult height; rhGH, recombinant human growth hormone; SDS, standard deviation score; TH, target height; Δ BMI, difference between BMI at last follow-up compared to baseline; Δ bone age/Δ chronological age, difference between bone age at last follow-up compared to baseline over difference between chronological age at last follow-up compared to baseline; Δ height, difference between height at last follow-up compared to baseline; Δ IGF-1, difference between IGF-1 at last follow-up compared to baseline

	**Total**	**dGHD**	**SUS**	**p**
N (%)	94 (100%)	26 (28%)	68 (72%)	
Age (years)	13.5 (11.1;15.1)	13.4 (10.8;14.5)	13.6 (11.3;15.4)	0.36
Length of treatment (years)	3.2 (1.4;4.1)	3.0 (1.2;4.1)	2.8 (1.6;3.8)	0.74
Height (SDS)	-0.9 (-1.8;-0.4)	-1.2 (-2.1;-0.6)	-0.9 (-1.8;-0.3)	0.21
Height – TH (SDS)	-0.5 (-1.4;0.1)	-1.2 (-1.7;-0.0)	-0.4 (-1.0;0.1)	**0.02**
Short stature (%)	18%	27%	15%	0.23
Δ Height (SDS)	0.9 (0.4;1.5)	0.8 (0.3;1.9)	0.9 (0.4;1.5)	0.54
BMI (SDS)	-0.3 (-1.1;0.5)	-0.5 (-1.4;0.5)	-0.3 (-1.0;0.6)	0.59
Overweight/obese (%)	10%/6%	15%/4%	7%/7%	0.43
Δ BMI (SDS)	-0.2 (-0.5;0.2)	-0.3 (-0.5;-0.0)	-0.2 (-0.5;0.4)	0.15
Bone age (years)	12.3 (9.0;14.0)	12.5 (8.9;13.6)	12.3 (10.0;14.4)	0.65
Bone age-chronological age (years)	-1.2 (-2.4;-0.3)	-1.0 (-1.8;-0.3)	-1.3 (-2.4;-0.4)	0.27
Δ bone age/Δ chronological age	1.0 (0.5;1.2)	1.1 (0.2;1.3)	0.9 (0.5;1.2)	0.82
IGF-1 (SDS)	-0.2 (-1.0;0.6)	-0.4 (-1.1;0.2)	-0.2 (-0.8;0.7)	0.34
Δ IGF-1 (SDS)	1.2 (0.1;1.9)	1.6 (0.2;2.8)	1.2 (0.2;1.9)	0.33
rhGH dose (mcg/kg/day)	28.1 (26.4;31.5)	29.8 (27.2;33.4)	27.8 (25.8;30.8)	**0.03**
